# Texture Analysis of T1-Weighted and Fluid-Attenuated Inversion Recovery Images Detects Abnormalities That Correlate With Cognitive Decline in Small Vessel Disease

**DOI:** 10.1161/STROKEAHA.117.019970

**Published:** 2018-06-01

**Authors:** Daniel J. Tozer, Eva Zeestraten, Andrew J. Lawrence, Thomas R. Barrick, Hugh S. Markus

**Affiliations:** 1 From the Stroke Research Group, Department of Clinical Neurosciences, University of Cambridge, United Kingdom (D.J.T., A.J.L., H.S.M.); 2 Neuroscience Research Centre, Molecular and Clinical Sciences Research Institute, St. George’s, University of London, United Kingdom (E.Z., T.R.B.).

**Keywords:** brain ischemia, cerebral small vessel disease, diffusion tensor imaging, magnetic resonance imaging, stroke

## Abstract

**Background and Purpose—:**

Magnetic resonance imaging may be useful to assess disease severity in cerebral small vessel disease (SVD), identify those individuals who are most likely to progress to dementia, monitor disease progression, and act as surrogate markers to test new therapies. Texture analysis extracts information on the relationship between signal intensities of neighboring voxels. A potential advantage over techniques, such as diffusion tensor imaging, is that it can be used on clinically obtained magnetic resonance sequences. We determined whether texture parameters (TP) were abnormal in SVD, correlated with cognitive impairment, predicted cognitive decline, or conversion to dementia.

**Methods—:**

In the prospective SCANS study (St George’s Cognition and Neuroimaging in Stroke), we assessed TP in 121 individuals with symptomatic SVD at baseline, 99 of whom attended annual cognitive testing for 5 years. Conversion to dementia was recorded for all subjects during the 5-year period. Texture analysis was performed on fluid-attenuated inversion recovery and T1-weighted images. The TP obtained from the SVD cohort were cross-sectionally compared with 54 age-matched controls scanned on the same magnetic resonance imaging system.

**Results—:**

There were highly significant differences in several TP between SVD cases and controls. Within the SVD population, TP were highly correlated to other magnetic resonance imaging parameters (brain volume, white matter lesion volume, lacune count). TP correlated with executive function and global function at baseline and predicted conversion to dementia, after controlling for age, sex, premorbid intelligence quotient, and magnetic resonance parameters.

**Conclusions—:**

TP, which can be obtained from routine clinical images, are abnormal in SVD, and the degree of abnormality correlates with executive dysfunction and global cognition at baseline and decline during 5 years. TP may be useful to assess disease severity in clinically collected data. This needs testing in data clinically acquired across multiple sites.

## Abstract 2

Supplemental Digital Content is available in the text.

Cerebral small vessel disease (SVD) causes lacunar stroke and is the major cause of vascular cognitive impairment and vascular dementia.^1^ The characteristic radiological features seen on magnetic resonance imaging (MRI) include lacunar infarcts, white matter hyperintensities (WMH), and cerebral microbleeds, which appear on different MR image types.

The normal appearing white matter (NAWM) in these images has been shown to be abnormal using advanced MR techniques, particularly diffusion tensor imaging (DTI).^2,3^ The degree of diffuse NAWM damage, assessed on DTI, has been shown to correlate more strongly with cognitive impairment than T2-WMH lesion load (WMHL).^4,5^ However, DTI is not routinely performed in clinical practice, and there is no simple measure that can be used to investigate NAWM damage on clinically acquired MRI.

One tool that could assess NAWM damage using conventional MR scans is texture analysis (TA). TA describes the relationship between the intensities of neighboring pixels and is particularly suited to tissue, such as NAWM, where there is only subtle disruption because of disease. This postprocessing technique has been used in T1- and T2-weighted images and magnetization transfer ratio maps.^6^

We hypothesized that TA might be sensitive to damage in the NAWM in SVD. Previous studies have shown correlations between NAWM DTI parameters and cognition,^3,7^ suggesting that NAWM damage is important in disease progression. Therefore, we applied gray-level co-occurrence (GLCM) matrix-based TA to conventional MR images of patients with symptomatic SVD and correlated the derived parameters with temporally equivalent neuropsychological test results. We also determined whether texture parameters (TP) were independent predictors of change in cognitive function in the same population and whether they could predict conversion to dementia.

## Methods

Because of subject confidentiality, the imaging data are not available to researchers. Summary data and analytical methods are available from the corresponding author on reasonable request.

### Subjects

Symptomatic SVD patients recruited to the SCANS study (St George’s Cognition and Neuroimaging in Stroke) were used in this study. This prospective longitudinal study recruited patients from a geographically contiguous region served by 3 hospitals in South London, United Kingdom.^3^ SVD was defined as a clinical lacunar stroke syndrome^8^ with an anatomically appropriate lacunar infarct on MRI, as well as confluent WMH (Fazekas grade ≥2) on MRI.^9^ The study was granted ethical approval by the Wandsworth research ethics committee (ref: 07/Q0803/82). All participants gave written informed consent. Participants were fluent English speakers, and MRI and cognitive tests were performed at least 3 months poststroke to minimize acute effects of stroke on cognition. Subjects underwent MRI and cognitive testing at baseline, then, of interest in this study, cognitive testing yearly for 5 years, 121 underwent MRI and cognitive tests at baseline; 99 of these attended for at least 1 follow-up visit, and details of the number of subjects at each time point can be found in the online-only Data Supplement.

A control population, imaged on the same MR scanner using the same sequences (for the last time point of the study only, which is used here^3^), comprised randomly selected community-dwelling subjects recruited to the GENIE study (St George’s Neuropsychology and Imaging in Elderly),^10^ these subjects had no history of psychiatric and central nervous system diseases (including stroke). There were data from 57 subjects available with mean±SD (age=70±9 years; 35 men); however, because of excessive movement or failure to tolerate the MRI protocol, data from 54 subjects were used.

### Clinical and Cognitive Performance Assessments (SVD Group Only)

All subjects had cerebrovascular risk factors recorded, including hypertension (systolic blood pressure of >140 mm Hg, diastolic blood pressure >90 mm Hg, or on treatment), diabetes mellitus (on drug or insulin treatment), hypercholesterolemia (random total cholesterol of >5.2 mmol/L or on treatment), body mass index, and smoking history (current, ex-smoker, and never).

Cognitive assessment was performed by a neuropsychologist using a battery of widely used tasks chosen to characterize the cognitive impairment seen in SVD,^3,11^ including digit-span, logical-memory, visual-reproduction, BIRT memory and information processing battery, speed of information processing, digit-symbol, grooved pegboard, trail-making test, verbal fluency, and modified Wisconsin card-sorting test. The following cognitive indices were constructed: executive function (EF), processing speed (PS), working memory, and long-term memory. A Global Cognition (GC) index was produced, which summarized performance on all tasks. Premorbid intelligence quotient was estimated using the restandardized National Adult Reading Test.^12^ Measures for each task were transformed into *z* scores through psychometric standardization using age-scaled normative data and averaged to calculate cognitive index scores.

Missing data from tasks which the subject was unable to complete because of the effect of cognitive decline, as judged by the attendant neuropsychologist, were addressed by substituting the minimum scaled score for that task (0 or 1; corresponding to *z* scores of −3.33 and −3). Other missing data were excluded from the calculation of cognitive index scores.

The slope of change in the cognitive data during 5 years was determined using linear mixed effects models to determine annualized change,^11^ and the cognitive parameters were modeled as a function of time allowing variation by fixed and random effects to determine linear trajectories and intercepts. The Wald test was used to determine whether there was a detectable change in the parameters over time for the average fixed effect slope for the group. Slopes were then estimated for each subject for the parameters showing significant change over time.

### Conversion to Dementia

Information on conversion to dementia during the 5-year follow-up was available for all 99 patients studied longitudinally. Dementia was diagnosed using the Diagnostic and Statistical Manual of Mental Disorders 5^13^ definition of major neurocognitive disorder and was present if individuals met one of the following:

1. A clinical diagnosis of dementia.
2. Review of medical records and cognitive assessments by a neurologist and clinical neuropsychologist, blinded to MRI and risk factor information, who agreed that the clinical picture met Diagnostic and Statistical Manual of Mental Disorders 5 criteria.
3. An Mini-Mental State Examination score consistently <24, indicative of cognitive impairment and reduced capabilities in daily living as measured by a score ≤7 on the instrumental activities of daily living.^14^

Date of dementia onset was defined as the date of diagnosis or the midpoint date between the visit at which the diagnosis was established and the previous visit.

### MRI Protocol

MRI was performed on a 1.5-T General Electric Signa HDxt MRI system (General Electric, Milwaukee, WI). The full MRI protocol has been described in detail.^3^

Sequences of interest to this study were as follows:

1. Axial fluid-attenuated inversion recovery (FLAIR): repetition time/echo time/inversion time=9000/130/2200 ms, field of view=240×240 mm^2^, matrix=256×192, 28 contiguous 5 mm slices.
2. Coronal spoiled gradient recalled echo T1-weighted: repetition time/echo time=11.5/5 ms, field of view=240×240 mm^2^, matrix=256×192, flip angle=18°, 176 contiguous 1.1 mm slices.

### Preprocessing and TA

Lesions were marked on the FLAIR images by an experienced rater, using the semiautomated lesion marking tools in DispImage.^15^ The T1-weighted images were segmented using SPM8 (Wellcome Department of Cognitive Neurology, UCL Institute of Neurology, London, United Kingdom), and the WM tissue probability map was lower thresholded at 90% to produce a WM segment. The T1-weighted images were registered to the FLAIR images using FLIRT,^16^ and the transformation applied to the WM segment. The lesion mask and WM segment were then applied to the FLAIR images to leave NAWM. The FLAIR images were registered to the T1-weighted images and the transform applied to the lesion masks to produce a T1-weighted NAWM map. For both registrations, 6 degrees-of-freedom were used to provide rigid-body transformation between the T1-weighted and FLAIR images.

The FLAIR and T1-weighted NAWM images were analyzed using the method proposed by Haralick et al,^17^ in which 14 TP are defined based on the GLCM: angular second moment, contrast, correlation, variance, inverse difference moment, sum average, sum variance, sum entropy, entropy, difference variance, difference entropy, information measure of correlation 1, information measure of correlation 2, and maximal correlation coefficient (MCC; for definitions, please see the online-only Data Supplement).

Because of the anisotropic voxel dimensions of the FLAIR images and to maintain methodological consistency between the image types, 2-dimensional TA was performed. The NAWM maps were normalized to a specified number of gray levels using histogram equalization. Based on the number of voxels in the NAWM segments and the number of gray levels in the native images, and to avoid issues with sparsity in the GLCM, the FLAIR images were normalized to 64 gray levels and the T1-weighted images to 128 gray levels. The GLCM and TP were calculated for the NAWM.

### Data Analysis

For analysis, the number of lacunes (nLAC) and number of microbleeds were log 10 transformed, and the T2-WMHL was expressed as a percentage of the total brain volume and log transformed.

Initially, TP from the NAWM were compared between the baseline SVD and control images using the Student unpaired *t* test.

Second, in the SVD cohort, the TP were correlated, using the Pearson correlation test, with normalized brain volume (NBV), WMHL, nLAC, number of microbleeds, and peak height from mean diffusivity NAWM (MDPH) histograms as determined previously to be the best MR-derived predictors of cognition.^3^

A regression analysis was then performed to determine whether TP were independently predictive of the cognitive measures. As in ref,^3^ univariate regressions were run for the TP, including age, sex, and premorbid intelligence quotient as confounds. For each image type, the variable showing the greatest standardized β (with associated *P*≤0.05) was chosen to progress to the multivariate model. These were included in a multivariate regression with the parameters listed above. Initially, only parameters derived from conventional MR images were added, and then a further model including MDPH was run. The regression tests were then repeated using the rates of change of the cognitive measures during the 5-year follow-up period.

Finally, the ability of TA to distinguish those of the SVD subjects who developed dementia was determined. Initially, univariate Cox regression was used to determine those TP that best predicted conversion to dementia (corrected for age, sex, and premorbid intelligence quotient), and these were then included in multivariate analyses as above, with addition of binary parameters indicating whether new microbleeds or lacunes are seen in follow-up scans, which were acquired for the first 3 years of the follow-up, to assess whether they are independent markers of conversion. To counter the possibility of overfitting, the model bootstrapping with 1000 samples of the population was applied.

## Results

### Subjects Excluded and Tissue Segments

Tissue segmentations were unreliable in 3 SVD subjects, and these were removed. Therefore, for the texture analyses, 118 subjects were included in the analysis. In a further 3 subjects, artifacts prevented reliable analysis of the DTI data, so for the regression, including the diffusion parameters, 115 subjects were included. Demographic details and MRI characteristics are shown in Table 1.

**Table 1.**
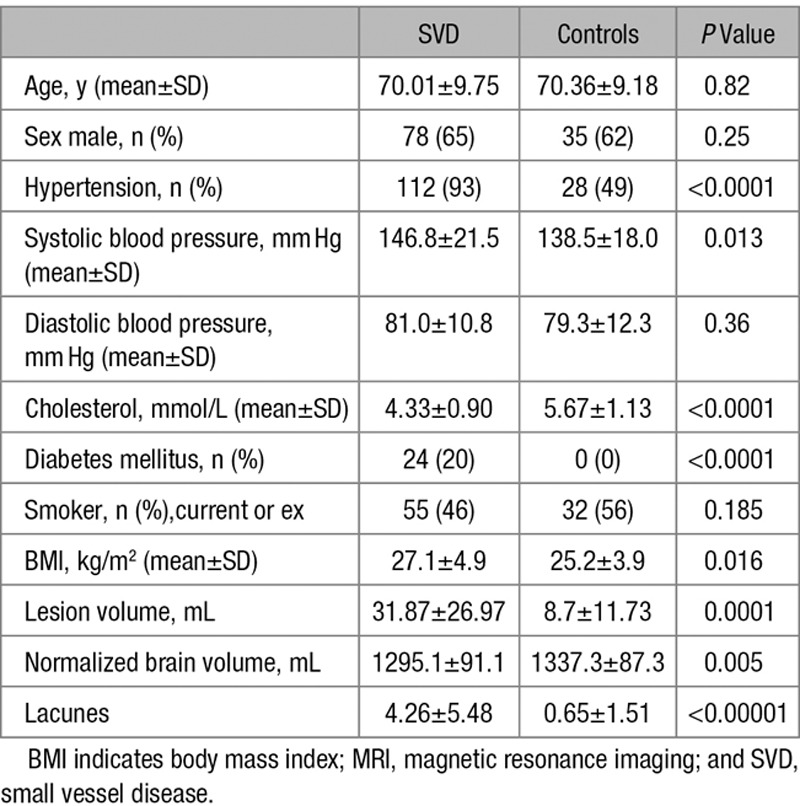
Group Demographics and MRI Characteristics for the SVD and Control Groups and the Results of the Group Comparisons

### Cognitive Change Over the Study Period

Details of the cognitive changes during the 5 years have been previously described,^11^ but in summary, PS, EF, and GC scores declined significantly during the 5-year follow-up period. The annualized changes were −0.048±0.015 (*P*=0.001) for EF, −0.052±0.014 (*P*<0.001) for PS, and −0.029±0.009 (*P*<0.001) for GC. It was, therefore, decided to proceed with the analysis using only these cognitive parameters.

### Cross-Sectional Analysis

#### Comparison of TP in NAWM Between SVD Cases and Controls

There were highly significant differences between SVD cases and controls for many TP derived from both T1 and FLAIR sequences (Table 2). The pattern of differences differed for the image types; only the sum entropy had *P*<0.0001 for both image types. The *P* values are not corrected for multiple comparisons because of shared variance between the measures. For reference, an uncorrected *P* value of 0.004 would give a Bonferroni-corrected *P* value of 0.05.

**Table 2.**
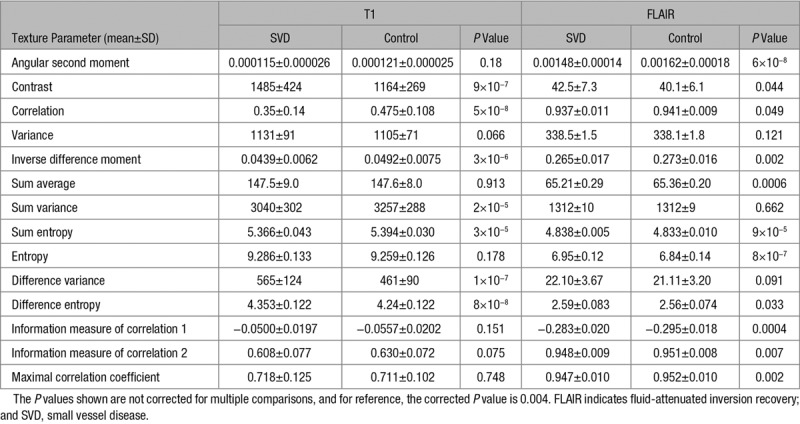
The Texture Parameters for the SVD and Control Groups for T1-Weighted and FLAIR Images and the P Value From the Independent Samples t Test Comparison

#### Correlation Between TP and Other MR Measures

The TP were highly correlated to several MR measures (Table 3), suggesting that the abnormalities in several different parameters underlie the changes in TP.

**Table 3.**
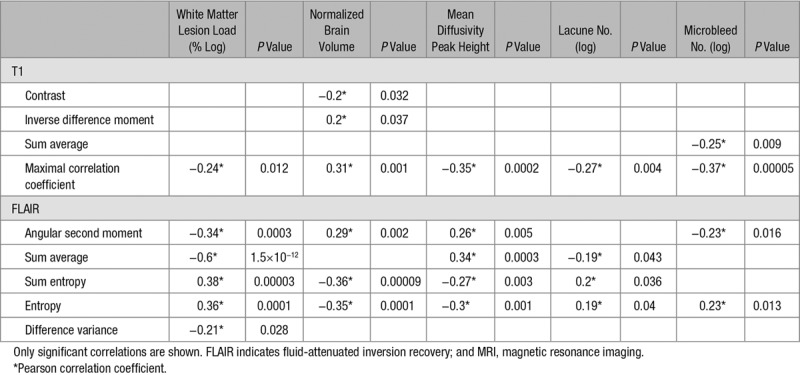
The Correlations of the Texture Parameters and MRI Measures

#### Regression Between TP and Cognition in the SVD Cases

As with previous work,^3^ only cognitive fields shown to change over the course of the study, and GC were included in the analysis.

For the univariate analysis in the EF, the T1-MCC (standardized β=0.182; *P*=0.009) and the FLAIR-sum average (0.196, 0.005) were the only significant regressors, only FLAIR-sum average significantly regressed with PS, and for GC, T1-MCC (0.188, 0.006) and FLAIR-sum average (0.24, 0.0005) were the most significant regressors. Results of the multivariate regression are shown in Table 4; FLAIR-sum average, WMHL, nLAC, and NBV associated significantly with EF, with associations being strongest for the texture parameter, nLAC, and WMHL. There was little difference whether MDPH was included in the model.

**Table 4.**
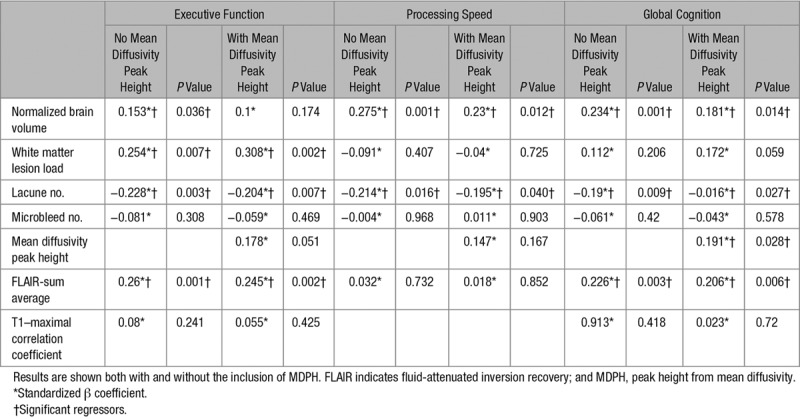
The Results of the Multivariate Regressions

Only nLAC and NBV independently correlated with PS. For the univariate regressions, no T1-weighted texture parameter was significant, and FLAIR-sum average was the most significant texture parameter (*P*=0.05), but this did not survive inclusion in the multivariate model. Again the addition of MDPH had little effect on the model.

The GC results were similar to those of EF, with NBV and nLAC being significant regressors along with the FLAIR-sum average, in this case, the MDPH also reached significance but did not change the results seen from the other parameters in the model.

### Longitudinal Analysis

#### Do TP Predict Cognitive Decline?

For the univariate regressions of the TP with the rate of change of the cognitive fields during 5 years, T1-MCC (standardized β=0.22; *P*=0.024) and FLAIR-entropy (0.312, 0.002) were significant regressors of EF, and the same parameters appeared for regression with GC (T1-MCC [0.205, 0.043]; FLAIR-entropy [−0.28, 0.005]); no TP had a significant regression with PS. When the other MR parameters are included, none of the TP remains significant, the closest to significance is for the change in EF without the MDPH, where FLAIR-entropy, a standardized β of −0.208 and a *P* value of 0.058. In addition, nLAC and National Adult Reading Test are significant for the GC change, whereas nLAC is significant for EF change.

For all regression models, the variance inflation factor was calculated and found to be <3 in all cases, suggesting no significant impact of multicollinearity.

#### Conversion to Dementia

Twenty of the 99 (20%) patients developed dementia during follow-up. Prediction of dementia was assessed by the Cox regression. On univariate regression, only 1 T1 image texture parameter was a significant predictor of conversion to dementia, the T1-MCC (*P*=0.042; hazard ratio [HR], 0.025). Several parameters from the FLAIR images predicted conversion to dementia, the best being FLAIR-sum average (*P*=0.002; HR, 0.106). In the multivariate bootstrapped model, the FLAIR-sum average retained significance (*P*=0.012; HR, 0.014) along with NBV (*P*=0.001; HR, 0.97), and when MDPH was also added, the same 2 parameters were significant in predicting dementia (FLAIR-sum average, *P*=0.006, HR, 0.008; NBV, *P*=0.001, HR, 0.971).

## Discussion

In this study, we demonstrated that TA, using standard MR sequences, detects abnormalities in NAWM structure in SVD compared with age-matched controls. Furthermore, TP correlated with EF and GC at baseline and predicted conversion to dementia, after controlling for age, sex, premorbid intelligence quotient, and other MR parameters. Of particular interest in this study are entropy, MCC, and sum average. Entropy describes the randomness of the gray-level pattern, the more random the pattern of gray levels in the image the greater the entropy. The sum average measures the sum of the diagonals in the GLCM, the more variation in the number of signal pairs the greater the value. Last, the MCC is the second principal component of the correlation matrix of rows of the GLCM, and it identifies variance not accounted for by the correlation parameter.

Our results are consistent with studies using different MR sequences, particularly DTI,^3,5,18^ which have demonstrated that the NAWM in patients with SVD is not normal, and its structural integrity correlates with cognitive impairment. What ultrastructural changes each of the individual TP are measuring is not clear, and it is likely that individual TP relate more closely to different underlying aspects of the pathology. However, within the SVD cohort, we found strong correlations between TP and brain volume, nLAC, WMHL, and atrophy, suggesting that the texture abnormalities are related to several different pathologies. These results support those seen in a recent article, where a relationship between TP with SVD score was seen.^19^

We found specific TP-predicted EF and GC at baseline and prospectively with longitudinal decline in both fields although this did not survive in the multivariate model. In contrast, TP did not correlate with PS or predict change in this parameter. This difference in relationships between executive dysfunction and PS is consistent with previous analysis of this data set in which diffuse WM changes were more closely related to EF^11^ while lacunar infarcts possibly acting through disruption of distributed brain networks were more closely related to impairment in PS.^20^

Although TP did correlate with cognition at baseline and predict decline in cognition, such correlations were only moderate and less strong than those with DTI parameters,^11^ where MDPH was a significant predictor of cognitive decline in the multivariate model. Furthermore, TP did predict conversion to dementia; however, the relationship was only of a similar order to the diffusion parameters which also showed a clear relationship with conversion to dementia (*P*=0.009; HR, 0.002).^11^ Also, the small number of subjects who converted to dementia means that the Cox regression may still be optimistic despite the use of bootstrapping. Thus, these results should be treated accordingly.

These results suggest that TA may be less sensitive to changes in NAWM ultrastructure than DTI, in particular, those predicting cognitive decline. However, TA has an advantage, particularly for large clinically acquired data sets, in that it can be performed on standard MRI sequences acquired during routine clinical scanning. TA produces a large number of highly correlated parameters. In this study, we selected those which were most strongly associated on univariate analysis to take through to multivariate analysis. Further replication studies are required to confirm that the parameters which we prioritized are most strongly associated with cognitive function in SVD in other populations. The third criterion for diagnosing dementia is not as clinically definite as the former 2, and this may lead to a misdiagnosis of some subjects; however, in the absence of a definite diagnosis, it was considered that those subjects showing this significant level of impairment should be considered as having dementia.

A major strength of this study is its longitudinal design with data on progression of cognitive decline during a 5-year period. A further strength is the inclusion not only of TP but also other MR parameters so that the independent contribution of TP to disease prediction could be determined. The SCANS cohort included patients with relatively homogenous SVD in that all had presented with lacunar stroke and all had confluent WMH. This is both a strength and a weakness of the study. However, further studies in cohorts of patients with different severity of SVD are required to determine whether these changes can be seen across the spectrum of SVD severity.

SVD presents a major health problem, and there are few proven treatments to prevent its progression. Recurrent stroke rates are relatively low, and it has been shown that neuropsychological testing is relatively insensitive to change both because of the slow rate of change and because of practice effects reducing the sensitivity to change.^21^ This has led to increasing interest in the use of MRI techniques to both monitor disease progression and test new therapeutic approaches in smaller phase 2 studies before large phase 3 trials with clinical end points.^22^ MRI markers, particularly DTI, have been shown to be potential surrogate markers being both sensitive to change over short time periods and able to predict future cognitive decline and dementia.^3,11^ However, despite an increase in availability, DTI is not acquired in all clinical MRI settings and takes additional time and image processing and can present challenges in multicenter studies. As TA can be performed on conventional MRI data, it has potential advantages in these situations. Our results suggest that TP may be useful markers of disease in SVD, but further studies are required to replicate these results and determine how TA performs in cohorts with multicenter imaging.

## Sources of Funding

The SCANS study (St George’s Cognition and Neuroimaging in Stroke) was supported by a Wellcome Trust grant (081589). Recruitment was supported by the English National Institute of Health Research (NIHR) Clinical Stroke Research Network. Prof Markus is supported by an NIHR Senior Investigator award, and his and Dr Tozer’s work are supported by the Cambridge University Hospital Comprehensive NIHR Biomedical Research Unit. Dr Lawrence is supported by a project grant from Alzheimer’s Research UK (ARUK-PG2013-2).

## Disclosures

None.

## Supplementary Material
