# Supplementary material for: Texture Analysis of T1-Weighted and Fluid-Attenuated Inversion Recovery Images Detects Abnormalities That Correlate With Cognitive Decline in Small Vessel Disease
Source: Stroke. 2018 Jun 1;49(7):1656–61. doi: 10.1161/STROKEAHA.117.019970 (PMC6022812; doi:10.1161/STROKEAHA.117.019970)
Supplement: Supplementary file 1 [file str-49-1656-s001.pdf]

Online Supplement

Daniel J. Tozer PhD[1], Eva Zeestraten PhD[2], Andrew J. Lawrence PhD[1], Thomas R. Barrick PhD[2], and Hugh S. Markus MD, PhD[1]

[1]Stroke Research Group, Department of Clinical Neurosciences, University of Cambridge

[2]Neuroscience Research Centre, Molecular and Clinical Sciences Research Institute, St. George's, University of London

Correspondence: Daniel Tozer

Box 83, R3 Neurosciences

Cambridge Biomedical Campus

Cambridge

CB2 0QQ

UK

Tel: +44(0)1223217718 Fax: +44(0)1223257288
E-mail: djt54@medschl.cam.ac.uk

Supplemental Methods

The 14 texture parameters derived from the grey-level co-occurrence matrix are defined below.

Each element (i, j) in the grey level co-occurrence matrix is given by *G(i, j)*, $G_x$ is the marginal probability matrix with elements as defined in (A.1) and $G_{x+y}$ and $G_{x-y}$ are as defined in (A.2) and (A.3), *N* is the number of distinct grey levels in the image.

$$G_x(i) = \sum_{j=1}^{N} G(i, j) \qquad \text{(A.1)}$$

$$G_{x+y}(n) = \sum_{i=1}^{N} \sum_{\substack{j=1 \\ i+j=n}}^{N} G(i, j) \qquad \text{(A.2)}$$

*for* $n \in \{2, 3, \ldots., 2N\}$

$$G_{x-y}(n) = \sum_{i=1}^{N} \sum_{\substack{j=1 \\ |i-j|=n}}^{N} G(i, j) \qquad \text{(A.3)}$$

*for* $n \in \{0, 1, \ldots., N-1\}$

TP1: Angular Second Moment (ASM):

$$f_1 = \sum_{i}^{N} \sum_{j}^{N} G(i, j)^2 \qquad \text{(A.4)}$$

TP2: Contrast:

$$f_2 = \sum_{n=0}^{N} n^2 G_{x-y}(n) \qquad \text{(A.5)}$$

TP3: Correlation:

$$f_3 = \frac{\sum_{i=1}^{N} \sum_{j=1}^{N} ijG(i, j) - \mu_x^2}{\sigma_x^2}, \qquad \text{(A.6)}$$

where $\mu_x$ and $\sigma_x$ are the mean and standard deviation of $G_x$.

TP4: Variance:

$$f_4 = \sum_{i=1}^{N} (i - \mu_x)^2 G_x(i) \qquad \text{(A.7)}$$

TP5: Inverse difference moment (IDM):

$$f_5 = \sum_{i=1}^{N} \sum_{j=1}^{N} \frac{G(i, j)}{1 + (i - j)^2} \qquad \text{(A.8)}$$

TP6: Sum average:

$$f_6 = \sum_{n=2}^{2N} nG_{x+y}(n) \tag{A.9}$$

TP7: Sum variance:

$$f_7 = \sum_{n=2}^{2N} (n - f_6)^2 G_{x+y}(n) \tag{A.10}$$

TP8: Sum entropy:

$$f_8 = -\sum_{n=2}^{2N} G_{x+y}(n) \log(G_{x+y}(n)) \tag{A.11}$$

TP9: Entropy:

$$f_9 = -\sum_{i=1}^{N}\sum_{j=1}^{N} G(i, j) \log(G(i,j)) \tag{A.12}$$

TP10: Difference Variance:

$$f_{10} = \sum_{n=0}^{N-1} (n - \mu_{x-y})^2 G_{x-y}(n), \tag{A.13}$$

where $\mu_{x-y}$ is the mean of $G_{x-y}$.

TP11: Difference entropy:

$$f_{11} = -\sum_{n=0}^{N-1} G_{x-y}(n) \log(G_{x-y}(n)) \tag{A.14}$$

TP12: Information measure of correlation 1:

$$f_{12} = \frac{f_9 + \sum_{i=1}^{N}\sum_{j=1}^{N} G(i, j) \log(G_x(i) G_x(j))}{-\sum_{i=1}^{N} G_x(i) \log(G_x(i))} \tag{A.15}$$

TP13: Information measure of correlation 2:

$$f_{13} = \sqrt{1 - e^{-2(H_{xy} - f_9)}}, \tag{A.16}$$

where $H_{xy} = \sum_{i=1}^{N}\sum_{j=1}^{N} G_x(i) G_x(j) \log(G_x(i) G_x(j))$

TP14: Maximal correlation coefficient:

$$f_{14} = \sqrt{\text{(Second largest eigenvalue of Q)}} \tag{A.17}$$

where $Q(i, j) = \sum_{k=1}^{N} (G(i,k) G(j,k)) / (G_x(i) G_x(k))$

These parameters describe how the signal intensities of neighbouring voxels are related. A moving window is applied to each voxel in the image and the relationship between the centre voxel and each of the surrounding voxels is considered. A GLCM is then created for each direction, for example the eight directions of the voxels immediately surrounding the centre voxel. These are then averaged to produce an overall GLCM. Descriptions of the meanings for some of the parameters can be found in Rekhil M Kumar et al. (IJCSIT) International Journal of Computer Science and Information Technologies, Vol. 5 (6) , 2014, 7668-7673, for example the ASM is high when the image is homogeneous, whereas the IDM is high when the image has high local homogeneity.

Supplemental table

| | Subjects with neuroimaging | Subjects with Cognition |
|---|---|---|
| Baseline | 121 | 121 |
| Year 1 | 94 | 96 |
| Year 2 | 74 | 77 |
| Year 3 | 68 | 72 |
| Year 4 | - | 37 |
| Year 5 | - | 59 |

Table I: Detail of the neuroimaging and cognition data available at each time-point in the study. Numbers include all subjects with neuroimaging and cognition at each time-point, prior to exclusions for image quality or other reason. Valid cognition numbers include sporadic missing cognitive data (3.86%) where individual tests were not be completed for various reasons, for example time constraints, patient motivation or experimenter error.

Five subjects did not have neuroimaging at year 1, but returned for year 2 and/or 3 of follow-up, giving a total of 99 subjects with multiple neuroimaging sessions. Similarly, seven subjects did not complete neuropsychological testing at year 1, but returned for (at least) one other testing session, giving a total of 103 subjects with multiple cognitive assessments.
